# Laparoscopic gastrectomy for elderly gastric-cancer patients: comparisons with laparoscopic gastrectomy in non-elderly patients and open gastrectomy in the elderly

**DOI:** 10.1093/gastro/goaa041

**Published:** 2020-09-10

**Authors:** Zheng-Yan Li, Jie Chen, Bin Bai, Shuai Xu, Dan Song, Bo Lian, Ji-Peng Li, Gang Ji, Qing-Chuan Zhao

**Affiliations:** 1 Xijing Hospital of Digestive Diseases, Fourth Military Medical University, Xi’an, Shanxi, P. R. China; 2 Department of General Surgery, Southwest Hospital, Third Military Medical University, Chongqing, P. R. China

**Keywords:** laparoscopic gastrectomy, elderly patients, gastric cancer

## Abstract

**Background:**

The benefits of laparoscopic gastrectomy (LG) in elderly gastric-cancer patients still remain unclear. The purpose of this study was to evaluate the feasibility and safety of LG in elderly gastric-cancer patients.

**Methods:**

We retrospectively evaluated patients who underwent LG or open gastrectomy (OG) between June 2009 and July 2015 in a single high-volume center. We compared surgical, short-term, and long-term survival outcomes among an elderly (≥70 years old) LG (ELG) group (*n *=* *114), a non-elderly (<70 years old) LG (NLG) group (*n *=* *740), and an elderly OG (EOG) group (*n *=* *383).

**Results:**

Except for extended time to first flatus, the surgical and short-term outcomes of the ELG group were similar to those of the NLG group. The ELG group revealed comparable disease-specific survival (DSS) rates to the NLG group (64.9% vs 66.2%, *P *=* *0.476), although the overall survival (OS) rate was lower (57.0% vs 65.5%, *P *<* *0.001) in the ELG group than in the NLG group. The ELG group showed longer operation time than the EOG group (236.4 ± 77.3 vs 179 ± 52.2 min, *P *<* *0.001). The ELG group had less estimated blood loss (174.0 ± 88.4 vs 209.3 ± 133.8, *P *=* *0.008) and shorter post-operative hospital stay (8.3 ± 2.5 vs 9.2 ± 4.5, *P *=* *0.048) than the EOG group. The severity of complications was similar between the ELG and NLG groups. Multivariate analysis confirmed that LG was not a risk factor for post-operative complications.

**Conclusions:**

LG is a feasible and safe procedure for elderly patients with acceptable short- and long-term survival outcomes.

## Introduction

Laparoscopic gastrectomy (LG) has been increasingly performed since it was first reported in 1994 by Kitano *et al.* [1]. Based on the experience accumulation of LG for early-stage gastric cancer, some experienced surgeons in high-volume centers have applied the laparoscopic procedure for patients with locally advanced gastric cancer (AGC) [2–7]. Recently, the results of several multicenter randomized–controlled trials (RCTs) showed that LG is a safe and feasible surgical procedure for AGC in short- and long-term outcomes [8–12].

With the rapid aging of the global population, the number of elderly patients has been continuously increasing. In China, elderly patients account for one-third of patients with gastric cancer [13]. Elderly patients are often considered a high-risk population for gastrectomy due to the high proportion of upper gastric cancer, advanced tumor stage, reduced functional reserve, and increased co-morbidities [14–17]. Therefore, elderly patients are always given more attention in the perioperative period than the non-elderly population. LG has been gradually accepted by more and more elderly patients, although the effects of pneumoperitoneum during LG are still in debate [18, 19]. To date, the feasibility of LG in elderly patients remains controversial [20, 21]. Research on the application of LG in elderly patients is lacking. To evaluate the safety and feasibility of LG in elderly patients, we compared the surgical and long-term survival outcomes with those for LG in younger patients and with those for OG in elderly patients.

## Patients and methods

### Patients

We selected patients who underwent LG and open gastrectomy (OG) at Xijing Hospital of Digestive Diseases, Fourth Military Medical University (Xi’an, China) between June 2009 and July 2015 in a prospectively maintained gastric-cancer database. As the OG was mainly performed before 2016, we limited the duration of study. Pathologic staging was updated according to the 8th Union for International Cancer Control (UICC)/American Joint Committee on Cancer staging system of gastric cancer [22]. The inclusion criteria for this study were as follows: pathologically confirmed gastric adenocarcinoma, an R0 resection, post-operative pathological stage I to III diseases, not combined with other malignancy, no prior surgery for gastric cancer, and no neoadjuvant chemotherapy. Finally, this study included 854 patients in the LG group and 383 elderly patients in the OG group. We categorized the patients by age: elderly (≥70 years old) and non-elderly (<70 years old). Among the patients who underwent LG, 114 patients were assigned to the elderly LG (ELG) group and 740 patients were in the non-elderly LG (NLG) group; 383 patients were in the elderly OG (EOG) group. This study was approved by the Institutional Review Board of our institution.

### Definition of co-morbidities

We divided the co-morbid diseases into seven categories: respiratory disease (chronic obstructive pulmonary disorder, interstitial pneumonia, asthma, spirometry abnormality), diabetes (controlled by medication or insulin), hypertension (controlled by medication), cardiovascular disease (coronary artery disease, cardiomyopathy, arrhythmia, past history of cardiac surgery or catheter intervention), cerebrovascular disease (brain infarction, neurodegenerative disease), liver disease (chronic hepatitis B/C, liver cirrhosis, hepatocellular carcinoma), and renal disease (chronic renal disease).

### Surgical procedures

The procedures for LG and OG have been previously described in detail [23–25]. All surgeries were performed by surgeons experienced in LG and OG. Patients chose the surgical types individually after they were informed of the surgical, complication, and oncological risks. We routinely administered post-operative adjuvant chemotherapy with 5-fluorouracil and cisplatin to each patient with stage II or more advanced cancer.

### Post-operative evaluation and follow-up

The primary endpoint was post-operative complication. The secondary endpoints were 5-year overall survival (OS) rate and 5-year disease-specific survival (DSS) rate. The OS period were defined as the interval between the date of operation to the date of death for any cause or the last follow-up. The DSS period was defined as the interval between the date of operation and the date of death due to gastric cancer or the last follow-up. Post-operative complications that occurred within 30 days after surgery were recorded and classified according to the Clavien–Dindo classification [26, 27]. Patients were followed up every 3 months during the first 2 years, every 6 months from 3 to 5 years, and then annually. The last follow-up date was July 2018.

### Statistical analysis

All statistical analyses were performed using SPSS, ver.22.0 (SPSS Inc., Chicago, IL, USA). The chi-square test was used to compare categorical variables and the independent sample *t*-test or Mann–Whitney *U* test was used to compare continuous variables. Multivariate analysis was conducted with the binary logistic-regression model to identify independent risk factors for post-operative complications. Survival curves were calculated using the Kaplan–Meier method and analysed by the log-rank test. All values were two-tailed and *P*-values <0.05 were considered significant.

## Results

### Clinicopathological characteristics

Table 1 summarizes the clinicopathological characteristics of the three groups. The ELG group showed higher ASA scores than the NLG group (*P *<* *0.001). Moreover, patients in the ELG group presented with more co-morbidity than those in the NLG group (*P *<* *0.001). No significant differences were observed between the ELG and the NLG groups in patient characteristics such as age, sex, body mass index, histological type, extent of resection, and TNM stage. The patient characteristics were comparable between the ELG and EOG groups.

**Table 1.** Baseline demographic and clinical characteristics of patients in the ELG, NLG, and EOG groups

Variables	ELG (*n* = 114)	NLG (*n* = 740)	*P*	EOG (*n* = 383)	*P*
Age^a^, years	74.2 ± 3.9	55.9 ± 8.5	<0.001	73.9 ± 3.4	0.455
Sex			0.470		0.801
Male	85 (74.6)	576 (22.2)		290 (75.7)	
Female	29 (25.4)	164 (77.8)		93 (24.3)	
BMI^a^, kg/m^2^	22.4 ± 3.2	22.8 ± 8.7	0.622	22.0 ± 3.0	0.190
ASA grade			0.001		0.768
1–2	67 (58.8)	661 (89.3)		231 (60.3)	
3	47 (41.2)	79 (10.7)		152 (39.7)	
Histological type			0.111		0.103
Differentiated	46 (40.4)	242 (32.7)		123 (32.1)	
Undifferentiated	68 (59.6)	498 (67.3)		260 (67.9)	
Extent of resection			0.686		0.196
DG	49 (43.0)	335 (45.3)		139 (36.3)	
TG	65 (57.0)	405 (54.7)		244 (63.7)	
pTNM stage			0.545		0.282
I	35 (30.7)	195 (26.4)		90 (23.5)	
II	32 (28.1)	204 (27.6)		125 (32.6)	
III	47 (41.2)	341 (46.1)		168 (43.9)	
Co-morbidity	41 (36.0)	125 (16.9)	0.001	126 (32.9)	0.543
Respiratory disease	29 (25.4)	31 (4.2)	< 0.001	91 (23.8)	0.807
Diabetes	12 (10.5)	44 (5.9)	0.066	34 (8.9)	0.727
Hypertension	15 (13.2)	35 (4.7)	< 0.001	58 (15.1)	0.708
Cardiovascular disease	10 (8.8)	17 (2.3)	< 0.001	31 (8.1)	0.970
Cerebrovascular disease	9 (7.9)	6 (0.8)	< 0.001	29 (7.6)	0.931
Liver disease	3 (2.6)	5 (0.7)	0.134	1 (3.4)	0.959
Renal disease	2 (1.8)	3 (0.4)	0.272	8 (2.1)	0.875
Other	2 (1.8)	4 (0.5)	0.400	7 (1.8)	0.959

a Except for these variables, other values are presented as numbers of patients followed by percentages in parentheses. ELG, elderly laparoscopic gastrectomy; NLG, non-elderly group; EOG, elderly open group; DG, distal gastrectomy; TG, total gastrectomy.

### Surgical outcomes

Table 2 shows the details of the surgical outcomes and post-operative complications of the three groups. The ELG and NLG groups showed no significant differences in operation time, estimated blood loss, number of retrieved lymph nodes, and post-operative hospital stay (all *P *>* *0.05). The time from operation to first flatus in the NLG group was shorter than that in the ELG group. Compared with the EOG group, the ELG group showed longer operation time (236.4 ± 77.3 vs 179.7 ± 52.2 min, *P *=* *0.001). The estimated blood loss (174.0 ± 88.4 vs 209.3 ± 133.8, *P *=* *0.008) and post-operative hospital stay (8.3 ± 2.5 vs 9.2 ± 4.5, *P *=* *0.048) were decreased in the ELG group compared with those in the EOG group. There were no significant differences in the number of retrieved lymph nodes between the ELG and NLG groups. However, the time from operation to first flatus was shorter in the ELG group than in the EOG group, although the difference was not statistically significant (3.9 ± 1.2 vs 4.2 ± 1.0, *P *=* *0.074).

**Table 2.** Surgical and short-term outcomes of patients in the ELG, NLG, and EOG groups

Variables	ELG (*n* = 114)	NLG (*n* = 740)	*P*	EOG (*n* = 383)	*P*
Operation time, min	236.4 ± 77.3	231.5 ± 76.1	0.523	179.7 ± 52.2	0.001
Estimated blood loss, mL	174.0 ± 88.4	169.8 ± 106.8	0.696	209.3 ± 133.8	0.008
Lymph-node dissection, *n* (%)			0.741		0.277
D1/D1^+^	21 (18.4)	127 (17.2)		89 (23.2)	
D2	93 (81.6)	613 (82.8)		294 (76.8)	
No. of retrieved lymph nodes	23.4 ± 7.7	24.3 ± 7.8	0.227	24.1 ± 9.7	0.440
Time to first flatus, days	3.9 ± 1.2	2.9 ± 1.4	<0.001	4.2 ± 1.0	0.074
Post-operative hospital stay, days	8.3 ± 2.5	7.5 ± 4.8	0.083	9.2 ± 4.5	0.048
Post-operative complication, *n* (%)					
Wound problem	2 (1.8)	12 (1.6)	1.000	13 (3.4)	0.538
Pulmonary complication	14 (12.3)	55 (7.4)	0.077	50 (13.1)	0.829
Intra-abdominal abscess	4 (3.5)	16 (2.2)	0.327	6 (1.6)	0.248
Intra-abdominal bleeding	2 (1.8)	15 (2.0)	1.000	8 (2.1)	1.000
Anastomotic leakage	5 (4.4)	19 (2.6)	0.354	8 (2.1)	0.187
Bowel obstruction	1 (0.9)	15 (2.0)	0.710	10 (2.6)	0.470
Hepatic	2 (1.8)	5 (0.7)	0.237	2 (0.5)	0.227
Cardiac	0 (0.0)	2 (0.3)	1.000	3 (0.8)	1.000
Overall complications (%)	20 (17.5)	89 (12.0)	0.130	83 (21.7)	0.340
Clavien–Dindo classification, *n* (%)			0.920		0.983
Grade II	14 (12.3)	65 (8.8)		56 (14.6)	
Grade III	3 (2.6)	14 (1.9)		12 (3.1)	
Grade IV	2 (1.8)	8 (1.1)		11 (2.9)	
Grade V	1 (0.9)	2 (0.3)		4 (1.0)	
Clavien–Dindo grade III/IV, *n* (%)	6 (5.3)	24 (3.2)	0.273	27 (7.0)	0.501

ELG, elderly laparoscopic gastrectomy; NLG, non-elderly group; EOG, elderly open group; D1 (TG: Nos. 1–7; DG: D1: Nos. 1–7); D1+ (TG: Nos. 1–8a, 9, 11p; DG: No. 1, 3, 4sb, 4d, 5, 6, 7, 8a, 9); D2 (TG: Nos. 1–8a, 9, 10, 11p, 11d, 12a; DG: No. 1, 3, 4sb, 4d, 5, 6, 7, 8a, 9, 11p, 12a).

### Post-operative complication

The overall post-operative complication rate in the ELG group did not differ from that in the NLG group (17.5% vs 12.0%, *P *=* *0.130). The severity of complications in the ELG group was also comparable to that in the NLG group in the severe-complication (Clavien–Dindo grade ≥IIIa) rate (5.3% vs 3.2%, *P *=* *0.273). We also observed no significant difference between the ELG and EOG groups in overall- and severe-complication rates (17.5% vs 21.7%, *P* = 0.340; 5.3% vs 7.0%, *P* = 0.501, respectively).

### Analysis of risk factors for post-operative complications

Multivariate analysis showed that pathological stage [odds ratio (OR), 1.854; 95% confidential interval (CI), 1.071–3.210, *P *=* *0.027] was an independent risk factor for overall complications after LG, but age was not (Table 3). For elderly patients, longer operation time (OR, 2.179; 95% CI, 1.206–3.937, *P *=* *0.010) and total gastectomy (OR, 1.714; 95% CI, 1.047–2.805, *P *=* *0.032) were independent risk factors for overall complications. Regarding severe complications, estimated blood loss >200 mL (OR, 3.208; 95% CI, 1.248–8.248, *P *=* *0.016) was identified as an independent risk factor in the LG group. Among elderly patients, longer operation time (OR, 2.734; 95% CI, 1.089–6.860, *P *=* *0.032) was shown as an independent risk factor for severe complications.

**Table 3.** Multivariate logistic-regression analysis of risk factors for post-operative complications

Variables	Overall complications				Severe complications			
	LG in elderly and non-elderly		LG and OG in elderly		LG in elderly and non-elderly		LG and OG in elderly	
	OR (95% CI)	*P*	OR (95% CI)	*P*	OR (95% CI)	*P*	OR (95% CI)	*P*
Surgical procedure, OG vs LG	—	—	0.778 (0.450–1.344)	0.368	—	—	0.754 (0.296–1.925)	0.555
Age, Non-elderly vs Elderly	1.480 (0.836–2.620)	0.179	—	—	1.583 (0.595–4.213)	0.358	—	—
Sex, Male vs Female	0.833 (0.502–1.382)	0.833	0.943 (0.558–1.595)	0.827	0.532 (0.181–1.562)	0.251	0.426 (0.144–1.262)	0.124
BMI, kg/m^2^ , <24 vs ≥24	0.989 (0.628–1.555)	0.960	1.411 (0.870–2.288)	0.827	0.970 (0.428–2.200)	0.942	0.800 (0.341–1.877)	0.608
ASA, 1–2 vs 3	1.106 (0.624–1.963)	0.730	0.999 (0.635–1.572)	0.998	0.988 (0.351–2.780)	0.982	1.428 (0.685–2.974)	0.342
Operation time, min, <240 vs ≥240	0.826 (0.436–1.564)	0.557	2.179 (1.206–3.937)	0.010	0.710 (0.195–2.588)	0.604	2.734 (1.089–6.860)	0.032
Estimated blood loss, mL, <200 vs ≥200	1.128 (0.734–1.733)	0.583	1.217 (0.767–1.931)	0.405	3.208 (1.248–8.248)	0.016	1.352 (0.622–2.937)	0.446
Histological type, Differentiated vs Undifferentiated	0.832 (0.534–1.297)	0.832	0.969 (0.605–1.552)	0.895	0.891 (0.394–2.012)	0.781	1.301 (0.582–2.909)	0.521
Extent of resection, DG vs TG	1.066 (0.694–1.638)	0.771	1.714 (1.047–2.805)	0.032	1.991 (0.849–4.671)	0.113	2.050 (0.868–4.840)	0.102
pTNM stage, I vs II–III	1.854 (1.071–3.210)	0.027	0.909 (0.538–1.535)	0.720	1.656 (0.588–4.661)	0.340	0.458 (0.207–1.012)	0.054
Co-morbidity, No vs Yes	1.290 (0.779–2.136)	0.322	1.349 (0.850–2.139)	0.204	1.466 (0.616–3.491)	0.387	2.528 (1.206–5.298)	0.014

DG, distal gastrectomy; TG, total gastrectomy; OR, odds ratio; CI, confidential interval.

### Long-term survival

**Figure 1.**
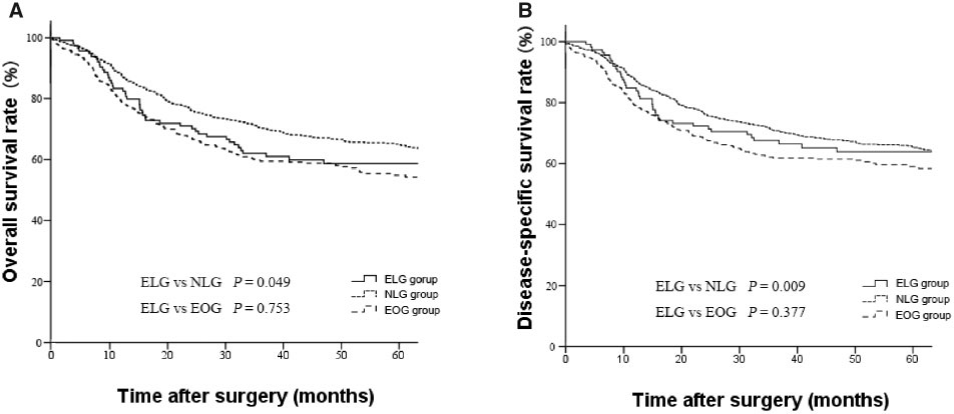
Kaplan–Meier OS and DSS curves for the ELG, NLG, and EOG groups. (A) OS; (B) DSS.

**Figure 2.**
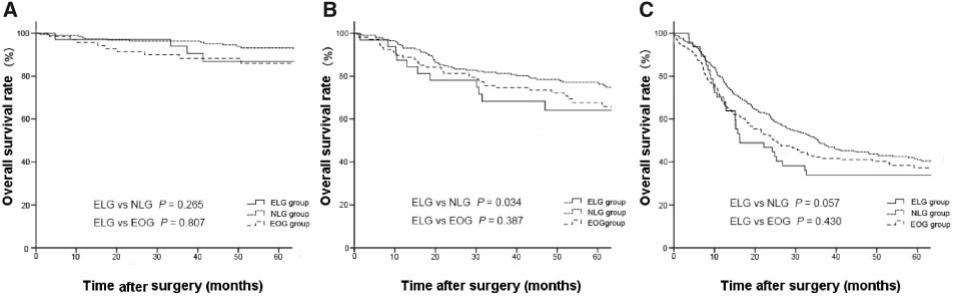
Kaplan–Meier overall survival curves for the ELG, NLG, and EOG groups. (A) Stage I gastric cancer; (B) stage II gastric cancer; (C) stage III gastric cancer.

**Figure 3.**
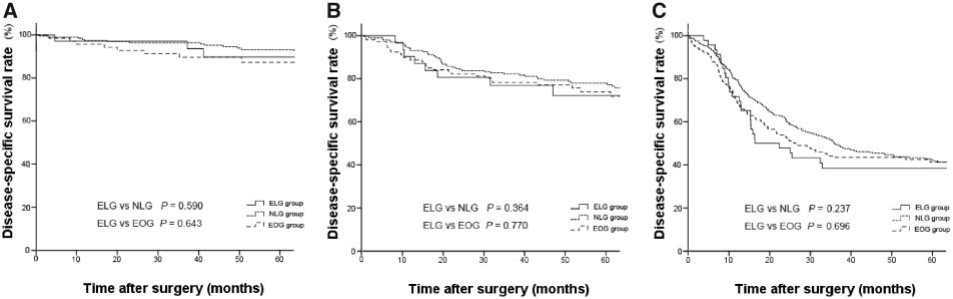
Kaplan–Meier disease-specific survival curves for the ELG, NLG, and EOG groups. (A) Stage I gastric cancer; (B) stage II gastric cancer; (C) stage III gastric cancer.

The 5-year OS rate was significantly lower in the ELG group than in the NLG group (57.0% vs 65.5%, *P *<* *0.001, Figure 1), whereas the DSS rate of the ELG group was similar to that of the NLG group (64.9% vs 66.2%, *P *=* *0.476, Figure 2). The 5-year OS and DSS rates were similar between the ELG and EOG groups (57.0% vs 56.7%, *P *=* *0.753, Figure 1A; 64.9% vs 60.6%, *P *=* *0.377, Figure 1B). The stage-specific analysis showed that the 5-year OS rate was significantly lower in the ELG group than that in the NLG group in stage II gastric cancer, whereas the 5-year OS rate of the ELG group was similar to that of the NLG group for stage I or III gastric cancer (Figure 2). Three groups showed similar DSS rates for stage I to III gastric cancer (Figure 3).

## Discussion

This study found that the short-term outcomes of the ELG group were better than those of the EOG group. Meanwhile, ELG group showed similar post-operative outcomes to those of patients in the EOG group in the incidence and severity of post-operative complications based on Clavien–Dindo classification. Moreover, no significant differences in the 5-year OS and DSS rates were found between the ELG and EOG groups.

Elderly patients are often considered a high-risk population for gastrectomy due to the high proportion of upper gastric cancer, more advanced tumor stage, reduced functional reserve, and increased co-morbidities [14–16]. Extensive research has shown that LG has the advantages of smaller incision, less bleeding, alleviated pain, and decreased surgical stress [28–30]. Elderly patients are at high risk of post-operative complications due to reduced functional reserve and increased co-morbidities. A recent meta-analysis demonstrated that elderly patients with gastric cancer who underwent LG had a higher overall post-operative complication rate than the non-elderly patients [31]. Kubota *et al.* [32] and Tokunaga *et al.* [33] found that post-operative complications had a negative effect on OS outcome even if the tumor underwent radical resection. We also found a similar phenomenon recently [34]. Moreover, our results showed that the negative effects were increased with higher Clavien–Dindo grades [35]. In the present study, although elderly patients tended to have higher ASA scores and more co-morbidities, the overall complication rate and severity of post-operative complications in the ELG group were similar with those in the NLG group.

Prolonged operation time and carbon-dioxide pneumoperitoneum during the surgical procedure are major concerns during LG for elderly patients, who exhibit higher rates of cardiovascular and pulmonary co-morbidities that can be adversely affected by longer anesthesia and pneumoperitoneum [9, 18, 19]. Longer operation time was always reported as a drawback of LG that acts as an negative factor for surgical outcomes [36]. In the current study, the mean operation time in the ELG group was significantly longer than that in the EOG group. A recent multicenter RCT reported that the mean operation time of the LG group was ∼30 min longer than that of the OG group, even when the surgeries were performed by experienced surgeons. Miki *et al.* [37] demonstrated that patients with longer operation time were associated with a higher risk of severe post-operative complications. In this study, multivariate analysis showed that extended operation time was an independent risk factor for the occurrence of severe complications in the elderly group. Additionally, LG was not identified as a risk factor for overall and severe complications, which is similar to the previous studies comparing the clinical outcomes of LG and OG for elderly gastric-cancer patients [20, 38].

Wang *et al.* [39] found that, for patients, combined pulmonary disease was a predictive factor for the occurrence of systemic complications after LG. Cho *et al.* [40] also reported that elderly patients with preoperative pulmonary diseases were associated with higher incidence of post-operative respiratory complications after LG. However, these results did not indicate that LG increases the incidence of post-operative respiratory complications for patients with preoperative pulmonary diseases. Suzuki *et al.* [18] found that cardiopulmonary impairment caused by carbon-dioxide pneumoperitoneum was transitory and could normalize during the intraoperative period. In the current study, our results showed that the pulmonary-complication rates between the ELG group and the EOG group were similar, which is consistent with the results reported in previous studies conducted by Zheng *et al.* [41] and Lu *et al.* [38].

To date, several studies have demonstrated that LG is a safe and feasible procedure for the treatment of elderly gastric-cancer patients [16, 21, 41, 42]. However, the primary endpoints of previous studies were mainly focused on short-term outcomes and therefore the long-term survival outcomes were seldom investigated. Regarding the effect of survival in elderly patients, Zheng *et al.* [41] reported similar 2-year OS rates between the LG and OG groups. Shimada *et al.* [20] demonstrated that LG could be a curative therapy procedure for elderly gastric-cancer patients in the comparison of 5-year DSS rates between elderly and non-elderly groups. In the current study, the 5-year OS rate of patients in the ELG group was lower than that in the NLG group. In China, the average lifespans of men and women are 74 and 77 years, respectively [43]. Based on this situation, elderly patients always died of causes other than gastric cancer. In this study, the DSS rate of patients in the elderly group was comparable to that in the non-elderly group. Moreover, the OS and DSS survival rates were similar between the two elderly groups. These results suggest that LG can be a safe procedure for elderly gastric patients in terms of long-term survival outcomes.

The number of retrieved lymph nodes is considered a key indicator of the quality of gastrectomy for gastric-cancer patients. Gastrectomy with D2 lymphadenectomy is standard in the treatment of locally AGC [44]. However, the reasonable extent of lymphadenectomy for elderly gastric-cancer patients still remains controversial. For these cases, some surgeons are usually reluctant to perform D2 resection because of the concerns of the potentially increased morbidity. Rausei *et al.* [45] found no significant benefits of D2 over D1 for patients >70 years old, although it showed the benefits in OS and DSS rates when the overall age band was taken into consideration. Takeshita *et al.* [46] demonstrated that limited lymph-node dissection had no negative effect for the DSS rates of elderly gastric patients. In this study, elderly patients with stage II or III disease account for 74.8% and a high percentage of D2 lymphadenectomies were performed. In contrast with several previous studies, our results showed no significant difference between the ELG group and the NLG group in the comparison of the number of retrieved lymph nodes and extent of lymphadenectomy. Previous studies with the purpose of evaluating the role of lymphadenectomy in elderly gastric-cancer patients had the limitations of small sample sizes and a retrospective nature. Therefore, large multicenter prospective RCTs should be conducted to further investigate the optimal extent of lymph-node dissection in elderly patients.

Several limitations of this study should be acknowledged. First, the post-operative complications are limited to grades II–V. This study did not consider complications in grade I, which was always not recorded in our clinical practice. Additionally, this is a retrospective study conducted in a high-volume center in China and all surgeries were performed by experienced surgeons, which may limit the applicability of our results to other centers and populations.

In conclusion, this study demonstrated that LG is a feasible and safe procedure for elderly gastric-cancer patients with acceptable short- and long-term outcomes. The results of this study need to be further validated by multicenter RCT studies.

## Authors’ contributions

ZYL, JC and QCZ designed the study. BB, SX and DS performed the research and retrieved the data. JPL, GJ and BL analyzed the data. ZYL and JC drafted the paper. All authors read and approved the final manuscript.

## Funding

This study was supported by the National Key Research and Development Program of China [2017YFC1311004] and The Key Research and Development Program of Shanxi province [2017-ZDXM-SF-053].
